# Remote Sensing of Atmospheric Optical Depth Using a Smartphone Sun Photometer

**DOI:** 10.1371/journal.pone.0084119

**Published:** 2014-01-08

**Authors:** Tingting Cao, Jonathan E. Thompson

**Affiliations:** Department of Chemistry & Biochemistry, Texas Tech University, Lubbock, Texas, United States of America; University of Aveiro, Portugal

## Abstract

In recent years, smart phones have been explored for making a variety of mobile measurements. Smart phones feature many advanced sensors such as cameras, GPS capability, and accelerometers within a handheld device that is portable, inexpensive, and consistently located with an end user. In this work, a smartphone was used as a sun photometer for the remote sensing of atmospheric optical depth. The top-of-the-atmosphere (TOA) irradiance was estimated through the construction of Langley plots on days when the sky was cloudless and clear. Changes in optical depth were monitored on a different day when clouds intermittently blocked the sun. The device demonstrated a measurement precision of 1.2% relative standard deviation for replicate photograph measurements (38 trials, 134 datum). However, when the accuracy of the method was assessed through using optical filters of known transmittance, a more substantial uncertainty was apparent in the data. Roughly 95% of replicate smart phone measured transmittances are expected to lie within ±11.6% of the true transmittance value. This uncertainty in transmission corresponds to an optical depth of approx. ±0.12–0.13 suggesting the smartphone sun photometer would be useful only in polluted areas that experience significant optical depths. The device can be used as a tool in the classroom to present how aerosols and gases effect atmospheric transmission. If improvements in measurement precision can be achieved, future work may allow monitoring networks to be developed in which citizen scientists submit acquired data from a variety of locations.

## Introduction

In recent years smart phones have become ubiquitous in society. These devices offer unique platforms for remote monitoring applications as they contain advanced processors and communication ability, are equipped with sophisticated sensors (cameras, accelerometers, and GPS), and are consistently located with an end user. In principle, the platform offers the potential of massively parallel sensing of environmental pollutants, inexpensive medical diagnosis, and unmatched utility for a variety of associated social and epidemiological studies. While it is unclear if this potential will ever be fully realized, several groups have already begun exploring the idea. Insurers have begun scoring consumers driving patterns based on acceleration and speed data collected with their smart phones [1]. A University group has used behavioral patterns assessed from data collected with smart phones to monitor physiological and mental health of a test group of students [2]. Smith *et al*. [3] have reported a simple cell-phone microscope and spectroscope fabricated from common laboratory supplies. A spectroscope and operating software has also recently been developed for pedagogical purposes [4]. In the research laboratory, Zhu *et al*. have used quantum dot luminescence as a probe to detect *E. coli* with cell phones [5]. Zhu *et al*. also have used a cell-phone to count fluorescently tagged cells flowing within a microfluidic channel [6]. In addition, Delaney *et al*. suggest cell phones coupled with electrogenerated chemiluminescence detection may offer options for rapid medical diagnosis in developing nations [7].

In this work we consider whether smart phone sun photometry is a viable option for remote sensing of atmospheric aerosols and gases. Our laboratory has an interest in developing single-point measurements to monitor the concentration and optical properties of airborne particulates (aerosols) [8]–[15] since particulate matter (PM) in earth's atmosphere degrades visibility, can affect climate, and human health. Particles in the micrometer size range effectively scatter electromagnetic radiation, so optical attenuation methods are suitable for estimating particle mass loading. However, single-point measurements can be costly to operate and maintain, require trained personnel to operate, and cannot provide air-column integrated information. In addition, information about the spatial pattern of aerosol loading cannot be obtained via one fixed sensor. In contrast, a network of smart-phone-based sun photometers operated by citizen-scientists could offer air-column-integrated information at many locations simultaneously. Indeed, recent efforts have been directed towards this goal (see http://ispex.nl/en/; accessed 10/31/2013).

Sun photometers measure the irradiance of sunlight within narrow wavelength bands that reaches earth's surface. The top-of-the-atmosphere (TOA) solar irradiance can be estimated through construction of a Langley plot, which is a calibration procedure that relates known changes in atmospheric path length (air mass) with observed changes in signals. Once the TOA intensity is known, atmospheric column transmittance (T) and integrated optical depth can be calculated. Changes of optical depth mostly depend on the quantity of light removed through scattering and absorption by aerosol particles, which is the reason why optical depth of cloudy days will be quite different from clear days. Previous authors have used sun photometry measurements to describe aerosol scattering or to measure the concentration of atmospheric gases such as O_3_ or water vapor [16]–[19]. In addition, measurements at multiple wavelengths has allowed retrieval of several aerosol characteristics including, size distributions, complex refractive index, and single-scatter albedo [20], [21]. In principle, if sun photometry data collected with a smart phone could quickly be sent to a central location and automatically processed, environmental monitoring could be conducted in nearly real-time on a continental-to-global scale. However, the quality of such data sets would be directly linked to the performance characteristics of the smart phone sun photometers used.

It is the purpose of this work to explore the use of several band pass filters and the camera of a smart phone as a sun photometer. We present data on the accuracy and precision of measurements achieved with a typical smart phone (iPhone 4) and use the phone for estimating direct surface solar irradiance in several wavelength bands. While the work described within focuses on an initial proof-of-concept and characterization study, a network of smart phone based sun photometers could be a valuable resource for monitoring atmospheric PM mass loadings, air quality, or UV index. Initial results suggest the smart-phone sensor could be a valuable tool for reporting air quality in areas heavily affected by pollution. The device is also attractive for use in an educational setting.

## Materials and Methods

### 2.1 Location, Solar Zenith Angle, and Airmass Estimation

All data was collected at ground level on the campus of Texas Tech University at Lubbock, TX (33.5778° N, 101.8547° W) during June - September 2012. The cosine of the solar zenith angle at any time/date for our location was computed by using the NOAA solar position calculator (http://www.esrl.noaa.gov/gmd/grad/solcalc/azel.html). Air mass was estimated as the inverse of this value. This estimate is known to be less accurate when the sun is near the horizon (air mass >10). Also, measurements made near sunrise/sunset are also subject to a higher uncertainty since air mass changes very rapidly at these times, and collecting data for a trial requires a finite amount of time. This introduces uncertainty into the Langley plot analysis described below.

### 2.2 Smartphone and Application Software

An iPhone 4 (Apple) was used for taking all measurements. The phones 8-bit, 5-megapixel iSight camera was used. Through experimentation, we found the smart phone camera produced some measureable response to light from a wavelength of approx. 200 nm to at least 1064 nm. In many Apps that run the camera, the ISO and shutter speed automatically adjusts based on ambient light level. This is clearly not acceptable for quantitative measurements so an application named 645 PRO (available in App store and Jag.gr) was used to take photographs since it allows the user control over variables such as exposure, ISO and focus. In order to avoid saturation of the 8-bit sensor, the maximum signal counts for each RGB channel must be <255. To achieve this we used neutral density filters to reduce the sunlight intensity and prevent potential damage to the camera. Then, the ISO of the camera was locked at 80 and the exposure time was at 1/10,000 sec. The white balance and auto focus was also locked. Also, Q-mode was chosen in the App to bypass the film mode. Through experimentation we found it was necessary to make sure the App was completely closed (not running in background) prior to starting it again for use. Failure to do this produces unreliable quantitative results, but the exact nature of this error is not fully understood. (Figure S1 in File S1 depicts the apparatus used for solar irradiance measurements.).

### 2.3 Optical Filtering & Laboratory Transmission Experiments

**Figure 1:**
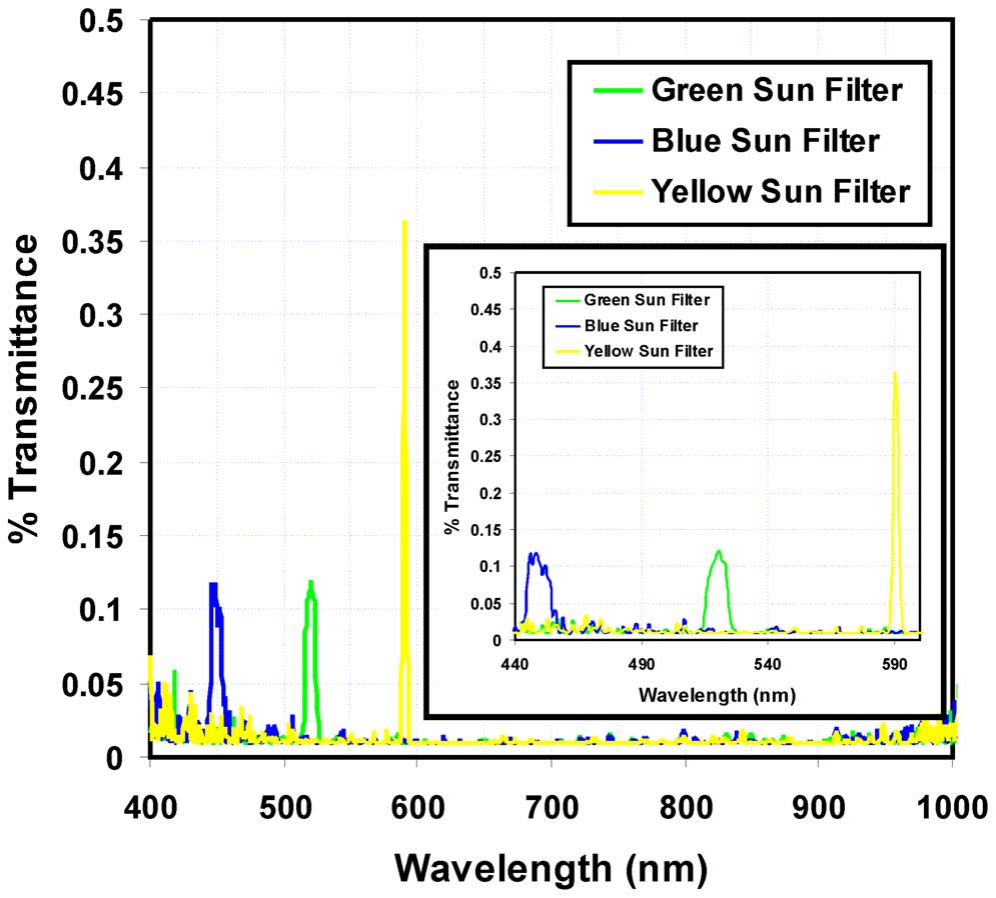
Plot of % Transmittance vs. wavelength for the 3 sun filters used in this study. Neutral density filters were used to attenuate the light to <1% of original value to provide “on-scale” measurements.

Three band-pass filters with the color of yellow (590±2 nm), green (520±10 nm) and blue (450±10 nm) were used to spectrally select sunlight by placing them directly in front of the iPhone camera lens. We refer to these filters as “sun filters.” Again, these sun filters were used to spectrally select the sun light. Neutral density filters were also used to attenuate the sunlight reaching the sensor. The transmission spectra of the sun filters are shown in Figure 1. The transmittances of the assembled filters (interference filters + neutral density) ranged between 0.1–0.4%. These low transmittances were needed to keep the cell phone camera sensor on-scale. Additional filters were also used to verify the accuracy of the sun photometer measurements made with the cell phone. For these experiments, four colored glass filters and four colored plastic filters were used. These filters are referred to as “test filters” and are essentially used to simulate aerosol loads (changes in transmittance). The test filters were used since their transmittances were fixed and known (or could be measured with a UV-VIS spectrophotometer) at the wavelengths of measurement. The glass test filters were a 400 nm long pass filter (FGL400), a 495 nm long pass filter (FGL495), a 550 nm short pass filter (FGL550), and a band pass filter (FGB37). The glass filters were obtained from Thor Labs, and a transmission spectrum for each filter is available at www.thorlabs.com. The colored plastic filters (LEE FILTERS) were named “straw tint”, “chocolate”, “dark steel blue”, and “surprise pink”. The transmission spectrum for each colored filter can be found at http://www.leefilters.com. The accepted percent transmittances of the test filters varied from near 0 to 92% at the test wavelengths. By comparing the smart phone measured transmittances for the test filters with their accepted transmittances, we can assess accuracy of the smart phone measurement. Smartphone measured transmittance was determined by taking photographs of the sun with and without a given test filter. These images corresponded to the test sample and spectroscopic blank (100% T), respectively. These experiments were conducted using the sun as the spectroscopic source, and a FGS 900 nm short pass filter was used to help eliminate near IR light which if present can lead to erroneous transmittance values. Since the atmospheric conditions can change during measurement it was decided that additional laboratory experiments should be performed in which a fiber-coupled UV-VIS light source (Hammamatsu, L10290) was used as the spectroscopic source. Details of the light source can be found in product information available at http://jp.hamamatsu.com/products/light-source/pd032/L10290/index_en.html. Light from the source was passed through the sun filters and into the smart phone camera. Again, the test filters provided samples of known transmission to test accuracy. It was anticipated the well-controlled laboratory setup would produce more accurate and precise measurements of test filter transmission, however, this was not achieved. Standard deviations of absolute errors (smart phone T – accepted T) were on the order of 0.06 or 6% transmittance. This is very similar to what was observed when the sun was the spectroscopic source. During experiments, we also found the order in which the filters were stacked to be significant. This is likely due to etaloning off multiple surfaces. The optimal order was when the sun filter was placed first in the optical path, followed by the IR blocking filter, and then neutral density and test filters.

### 2.4 Image Analysis & Data Processing

All photos were imported into a personal computer and analyzed with Image J software (http://rsbweb.nih.gov/ij/). This allowed RGB count levels for each image pixel to be determined. For this procedure, the image file was opened and cropped to an area that depicted the center of the sun. An area was selected and the mean RGB intensities determined. This data was used without any further corrections for the subsequent calculations. Other areas in the photographs that did not depict the sun appeared very dark (e.g. black) and had very low signal counts (generally <<2 cts) so there was not a significant dark signal. If present and unaccounted for, dark counts could cause error in quantitative analysis. For accuracy experiments conducted with the test filters, the mean RGB values for blanks were assumed as 100% transmittances, and the percent transmittances of all test filters were calculated according to the ratio of the RGB channel values when the test filter was in the beam compared to the blank values. For ambient measurements of optical depth, the observed smart phone sun photometer signal counts (S*_observed_* - 0 – 256 counts) were determined in Image J and then ratioed to the expected top-of-the atmosphere (S*_TOA_*) signal values for each channel obtained via the Langley analysis described in section 3.2. This is the expected signal that would be obtained if the sensor was placed at the top of the atmosphere. Taking the ratio of S_observed_/S_TOA_ resulted in calculation of atmospheric transmission. Measured atmospheric optical depth (τ) was then computed via:
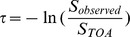



For the stratus cloud data the Angstrom exponent was determined by plotting the observed optical depths vs. wavelength of measurement (using all 3 wavelengths) and fitting the data to a power law. The exponent value was rejected from the data pool if the fit to power law yielded an R^2^<0.75. Most R^2^ values encountered were >0.9.

## Results and Discussion

### 3.1. Accuracy and Precision of Smartphone Measurements

The precision of replicate measurements was assessed by studying the variability in replicate blank measurements with only the sun filters present in the optical path. The image-to-image reproducibility was good, with an observed relative standard deviation of 1.2% in observed counts (28 trials with 145 total data points). From this we can estimate a minimum detectable transmission change as 3 times this value or a ΔT = 3.5%. This corresponds to an optical depth of roughly 0.04. To put this in perspective, the National Oceanic and Atmospheric Administration (NOAA) states typical aerosol optical depths for the United States is approximately 0.1–0.15 [22]. These values suggest smart phone sun photometry should be a viable option.

**Figure 2:**
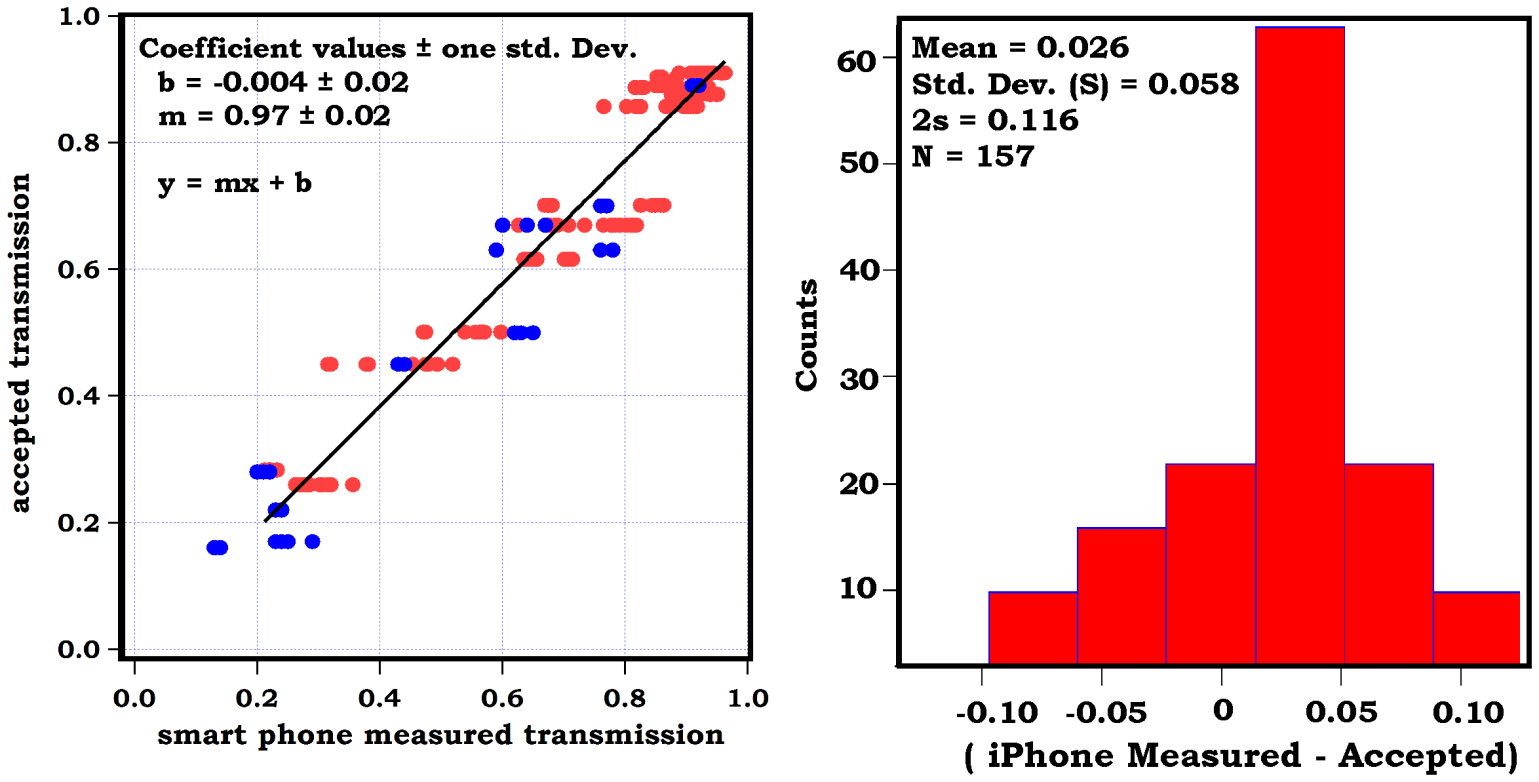
Evaluation of Accuracy. *Left* – Plot of accepted transmittance of a series of optical filters *vs*. smart phone measured transmittance using the sun (red) and a lamp (blue) as light source. The accepted filter transmittances were determined by either product literature or by using a spectrophotometer. The solid best-fit line was determined by orthogonal distance regression. The slope of the line is close to unity and the intercept indistinguishable from zero indicating good agreement between the measurements. *Right* – Histogram of differences between smart phone measured and accepted transmittances for sun data. A slight positive bias is reflected in the data. The calculated standard deviation (*s*) of differences between measurements was 0.058, yielding a 2*s* uncertainty of ±0.116 or 11.6% T.

To investigate further we have performed experiments to assess the linearity and accuracy of the smart phone response. To accomplish this we have used the smart phone to make transmittance measurements of several optical filters of known transmission. We can then compare measured and accepted values directly. Results of this experiment are shown in Figure 2. As observed, the best-fit line determined by orthogonal distance regression has a slope close to 1 and an intercept near zero. This suggests the smart phone measured transmittance is in agreement with the accepted values and no large error is present in the measurement on average. However, measurement data is clearly scattered above and below the 1∶1 line and this suggests significant variability/imprecision. We originally believed this scatter may be due to variability of aerosols in the atmosphere that we could not account for. However, the laboratory experiment using a lamp as a light source was not able to improve the measurement precision compared to solar measurements. To analyze this further we have prepared a histogram of the differences between iPhone measured filter transmittances and the accepted values (also presented in figure 2). As observed, most measured values are within 5% of the accepted transmittance, although a very slight bias (smart phone slightly high) may be apparent in the data. The standard deviation of the differences (iPhone%T – Accepted%T) was 0.058. If we assume the data is Gaussian distributed and 0.058 is representative of the standard deviation, we can define a 2s criteria for defining uncertainty. This criteria and the standard distribution leads to the conclusion that roughly 95% of replicate smart phone measured transmittances should lie within ±11.6% of the true value. This converts into an uncertainty in optical depth of roughly 0.12–0.13. We advocate these values for the uncertainty associated with the measurements made with the smart phone. This uncertainty is comparable to the mean aerosol optical depths for the continental United States cited previously, and is significantly poorer performance (by at least 1 order of magnitude) than commercially available sun photometers. For instance, the handheld Microtops II sunphotometer (solarlight.com) offers a precision of 1–2%. Also, the AERONET program estimates an overall uncertainty in optical depth of 0.01–0.02 for the research grade sunphotometers they employ (CE-318 CIMEL) [23]. Thus, it appears the smart phone sensor we describe here is best suited for environments in which optical depth is relatively high. This could correspond to applications in which users rapidly report significant dust events or use the smart phone sun photometer to monitor air quality in highly polluted urban centers.

### 3.2. Computation of Langley Plots

**Figure 3:**
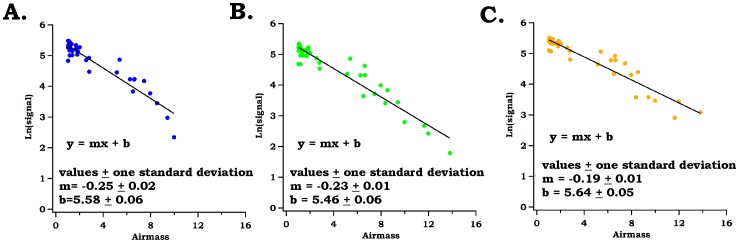
Langley Plots (ln signal counts *vs*. air mass) for the blue (A), green (B), and yellow (C) sun filters. The slope of the best-fit lines represents optical loss per unit air mass while the intercept describes the signal expected at the top-of-the-atmosphere (TOA). Best-fit lines were determined by orthogonal distance regression.

A significant problem in resolving integrated atmospheric optical depth is determining the signal a sensor would measure at the top-of-the-atmosphere (spectroscopic blank or 100% T). This is usually accomplished through an ingenious method known as Langley extrapolation [24]–[25]. In this approach, the detector signal for solar irradiance in a given wavelength band is logged at earth's surface throughout a day during which the sky is clear, humidity is low, and conditions are constant during the experiment. The solar zenith angle changes throughout the day, meaning the path length through the atmosphere differs from sunrise to sunset. This leads to differences in air mass (m, *e.g*. path length). When the sun is directly overhead (zenith angle  = 0), the air mass is equal to m = 1. At dusk and dawn, air mass can exceed 15. If attenuation is caused by a homogeneous atmosphere, following the Beer-Lambert law we can write:
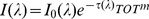



Where *I(λ)* and *I_0_(λ)* are irradiances at a given wavelength at the surface and the top of the atmosphere, *m* is air mass, and τ(λ)*_TOT_* is the optical depth *per-unit-airmass* at this wavelength. Rearranging Eqn. 2 to create a new calibration yields:
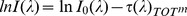



Equation 3 suggests a linear relationship exists between *ln I(λ)* and air mass (m) if all other variables are held constant. If the detector response is linearly related to surface irradiance, then a plot of *ln* (observed signal counts) *vs*. air mass (m) should yield a line. The y-intercept of this line represents *ln I_0_ (λ)* which can yield the expected signal at the top of the atmosphere in a specific wavelength band. Figure 3 illustrates Langley plots for the cell phone sun photometer on the blue, green, and yellow channels. In order to generate these plots photos were taken every hour from sunrise to sunset on several days during July 30 – August 4 2012. These days featured clear and cloudless skies with temperature maxima of 35–40°C. Light winds from the southwest at 5–10 mph were typical. Air mass was computed from our locations geographic coordinates as described in the methods section. As observed in the figure, plots illustrating linear trends were obtained (R^2^ approx. 0.9). The slopes of the best-fit lines were −0.25, −0.23, and −0.19 for the blue, green and yellow channels, respectively. This ordering is consistent with the enhancement in Rayleigh scattering at blue wavelengths; however, the observed slopes are not quantitatively consistent with the well-known λ^−4^ trend since these slopes are indicators of the total optical depth per unit airmass. Clearly, attenuation processes involve both gas and aerosol scattering (aerosol scattering does not follow the λ^−4^ trend). Also, the camera response may vary with wavelength.

### 3.3. Monitoring Changes in Optical Depth

**Figure 4:**
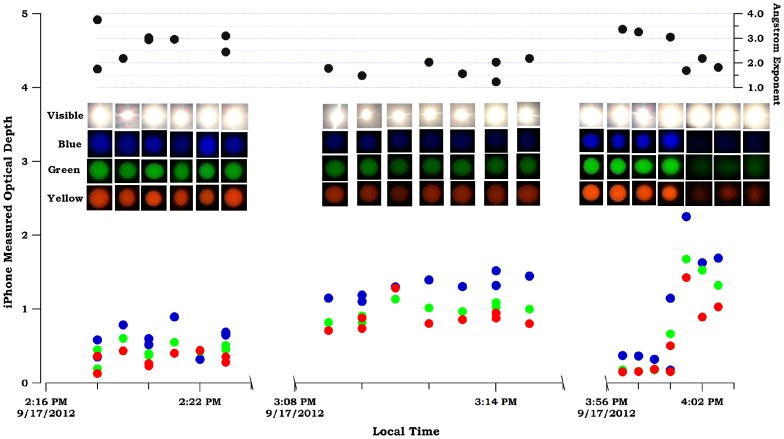
Monitoring atmospheric optical depth and Angstrom exponent at Lubbock, TX during the afternoon of September 17 2012 using the smart phone sun photometer on the blue, green, and yellow channels. This afternoon featured a very thin, high-altitude stratus cloud layer that periodically blocked the sun and increased optical depth. This effect is particularly noticeable near 4:00 PM local time when a rapid and large increase in optical depth was observed. This change was accompanied by a reduction in Angstrom exponent by approx. 1 unit which suggests larger particles contribute to the increase in optical attenuation. Angstrom exponents reported consider the effect of both gases and particles. No measurements are made on the “visible” channel – this is only included to provide the reader with a visual reference.

After constructing the Langley plots for each wavelength, we turned our attention to monitoring the optical depth of the atmosphere using the smart phone device and sun filters. The afternoon of September 17 2012 provided an excellent opportunity to monitor solar attenuation since a high-altitude and thin stratus cloud layer was present at our location. Photographs of the sun were sequentially acquired through each of the three interference filters. Indicated signal counts were ratioed to the top-of-the-atmosphere signal obtained via the Langley analysis and optical depth computed from the resulting atmospheric transmittance. Measured optical depths were then plotted in time as reported in figure 4. Figure 4 also plots observed Angstrom extinction exponents (AEE) values in time. The Angstrom exponent (α) is an empirically derived value used to describe optical attenuation with wavelength [26]. The basic premise is that the trend in atmospheric optical attenuation with wavelength follows a power law of the form: 
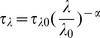



Where the τ terms represent optical depths at two different wavelengths, and λ and λ_0_ represent the two wavelengths considered. The Angstrom exponent (α) essentially describes how rapidly attenuation changes with wavelength. It can reach a maxium value of α = 4 for pure Rayleigh scatterers (gas molecules) and can be near zero (or even negative) for very large particles [27], [28]. This is in fact why the clear sky appears blue but a overcast sky appears white or grey. Considering Figure 4 we can easily see the influence of the stratus clouds on observed optical depth around 4:00 PM local time as the optical depth increases from <0.5 to 1.5 over a few minutes. Interestingly, the Angstrom exponent observed suddenly decreases from approx. 3 to about 2 simultaneously. This decrease in average α is consistent with micron-sized particles such as water drops or ice influencing optical attenuation. In addition, for the middle and left panel of Fig. 4, we see highest Angstrom exponents were generally observed when optical depth was lowest. This is consistent with airborne particles being responsible for increasing the change in optical depth sensed.

### 3.4 Comparison of Sun Photometer with a Reference Device

**Figure 5:**
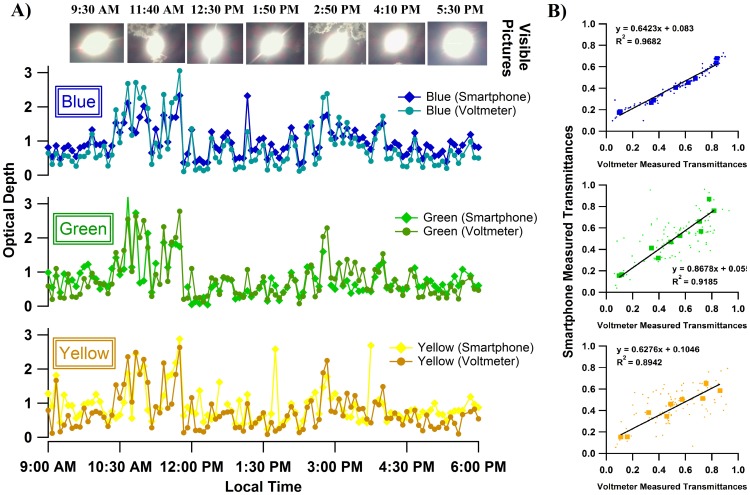
Optical depths measured with smartphone sun photometer and reference device. Panel (A) – Optical depths at Lubbock, TX from 9:00 AM to 6:00 PM on September 1 2013. Measurements were made with both smart phone sun photometer and reference method on blue, green and yellow channels. On this day, optical depths shifted up and down because of the existence of clouds in the sky as seen in the photographs. (B) – Comparison of transmittance measurements using smartphone and reference method. The y-axis shows atmospheric transmittance measured by the smartphone while x-axis shows transmittance measured by reference method. Small dots illustrate all measurements made with the two devices. Larger square markers are median values for different bins. Best-fit lines were determined by orthogonal distance regression from medians.

Unfortunately, we did not have access to a commercial sun photometer during our study to compare measurements. Instead, a comparison measurement was made using a Si biased detector (Thorlabs, DET36A) integrated with a voltmeter (GB Instruments, GDT-11) and the three interference filters (sun filters) described earlier. The field of view for the reference device was approx. 5 degrees. Linear response for this sensor was verified and Langley plots were constructed separately for this detector (see Figure S3 in File S1 and Fig. S4 in File S1) so that optical depths could be measured and compared for both the reference method, and smartphone. Measurements with both smartphone and reference method were taken on September 1 2013 from 9:00 AM to 6:00 PM local time. On that day, clouds were present in Lubbock, TX that periodically blocked the sun. Optical depth changes for both the smartphone and reference method are plotted in Figure 5. To ensure the accuracy of measurements, we performed an additional Langley calibration with the smartphone during August 22–24 2013 (see results in Figure S2 in File S1). Trends of optical depths measured with smartphone were generally consistent with the result from the reference device, for blue, green and yellow channels. During the whole day, optical depths shifted up and down showing the effects of the cloud layers in the sky. Clouds reflect and may absorb light from the sun, resulting in reduction of solar irradiance onto the surface of the earth. During 10:30 AM – 12:00 PM and 2:40 to 3:00 PM, optical depths went up to 2–3, indicating the existence of optically thick clouds in the atmosphere. These results were in agreement with the visible pictures shown at the top of Figure 5A indicating clouds were present. In Figure 5B, we plot the transmittance results obtained from both devices to directly compare them. Small dots illustrate all measured data points for the two devices. The larger square markers represent the median values when data was sorted by value and binned. From the best-fit lines determined by linear regression, we got the slopes of 0.64, 0.86 and 0.63 (R^2^ approx. 0.9) and intercepts near zero. The data of Fig. 5B suggests a systematic underestimation of transmittance for the smartphone. The exact cause of this is unclear. The underestimation could be the lack of accuracy for the device itself; however, asynchronous measurements can also lead to variation between the two devices. Nonetheless, the measurements produced by the two separate devices are proportional to one – another.

## Conclusions

A common smart phone has been adapted for use as a sun photometer. The uncertainty in optical depth achieved is at least on the order of 0.12. This is approximately an order of magnitude poorer performance compared to research grade devices, but this disadvantage is offset to a degree by the ubiquity of smartphones. The measurement uncertainty is also similar to the average aerosol optical depths for the continental United States. These results suggest improvement in quantitative performance will be required before the full potential of the smart phone sun photometer can be reached for environmental research. Nonetheless, this work has demonstrated the concept may be viable – particularly during periods of high aerosol loading such as in dust events or in polluted urban centers where aerosol optical depths often exceed 0.12. We have clearly demonstrated a change in observed optical depth during the transit of a thin cloud through the optical path between the sun and an observer. This increase in optical depth corresponded with a decrease in Angstrom extinction exponent. This would be expected for optical attenuation by large water or ice particles of a cloud.

Further investigations should focus on defining the limit of spectroscopic precision practically achievable with the on-board camera. The 8-bit resolution of the cameras plays a role in defining this limit, however, our current measurement uncertainty is >20 fold worse than what we might expect based on the 8-bit resolution alone. Significant performance improvement could be achieved by building a stand – alone spectroscopic sensor that would plug into the smart phone, however, this would significantly decrease user friendliness and could add significant additional expense to a monitoring network. In addition to the hardware limitations, new smart phone application software (an app) should be written to serve the specific purposes of the experiment. Data analysis was time consuming and complicated. In principle, it should be possible to author an app that could collect data and analyze results rapidly in the field. This could include use of geospatial data and the clock capability to rapidly determine air mass. Geospatial data could also be used for rapid and automated spatial mapping of results obtained. In summary, creating a network of smart phone sun photometers for real time chemical and aerosol monitoring is possible provided measurement uncertainty can be reduced and a suitable app created that maximizes measurement precision while automating data analysis.

## Supporting Information

File S1 Supporting file.
(PDF)
