# Supplementary material for: Remote Sensing of Atmospheric Optical Depth Using a Smartphone Sun Photometer
Source: PLoS One. 2014 Jan 8;9(1):e84119. doi: 10.1371/journal.pone.0084119 (PMC3885532; doi:10.1371/journal.pone.0084119)
Supplement: File S1 — Supporting file. (PDF) [file pone.0084119.s001.pdf]

Supplementary Information:

Remote Sensing of Atmospheric Optical Depth Using a Smartphone Sun Photometer

Tingting Cao and Jonathan E. Thompson*

[1] Department of Chemistry & Biochemistry, Texas Tech University, MS1061, Lubbock, TX 79409; E-Mail: jon.thompson@ttu.edu

* Author to whom correspondence should be addressed; E-Mail: jon.thompson@ttu.edu; Tel.: +1-806-742-3210; Fax: +1-806-742-1289.

1. Experiment Setup

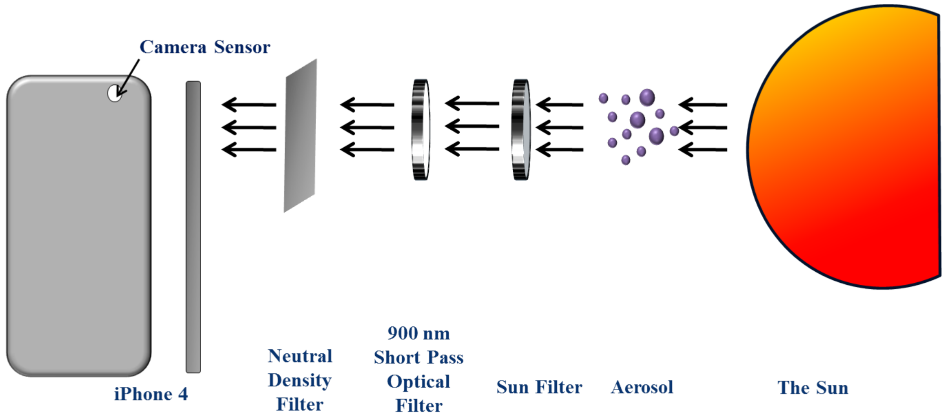

Fig. S-1 Instrument setup of smartphone sun photometer

As depicted in Figure S-1, three sun filters with the color and narrow band wavelength of yellow (590 ± 2 nm), green (520 ± 10 nm) and blue (450 ± 10 nm) were used to spectrally select sunlight by placing them directly in front of the iPhone camera lens. Neutral density filters were used to attenuate the sunlight reaching the sensor for camera protection and to ensure the signals were "on-scale". A FGS 900 nm short pass filter was used to help eliminate near IR light which if present can lead to erroneous transmittance values. As the concentration of aerosol particles in between the filters and the sun changes, the optical depth of the atmosphere will also change. This can be observed and monitored. The sun was tracked by manually pointing the smartphone device and centering the image of the sun on the screen.

2. Calibration of Smart Phone Sunphotometer During August 22-24 2013

During August 2013 we attempted a series of comparison experiments of the smartphone sun-photometer with an alternate device. This required construction of an additional Langley plot as shown in Figure S-2. Procedures were the same as described in the paper.

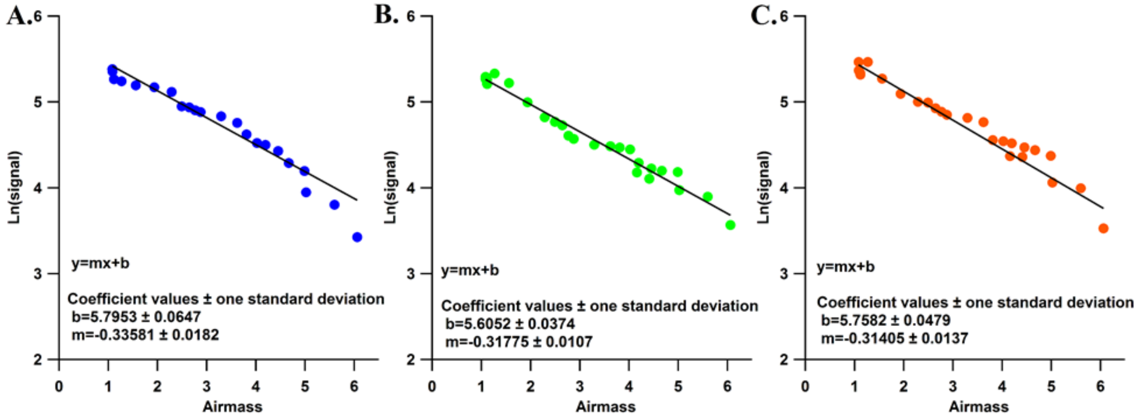

Fig S-2. Langley Plots (ln signal counts vs. air mass) measured by smartphone for the blue (A), green (B), and yellow (C) sun filters. Best-fit lines were determined by orthogonal distance regression.

Figure S-2 illustrates Langley plots for the cell phone sun photometer on the blue, green, and orange channels. Photos were taken during August 22 - August 24 when air mass changed from approx. 1 to 6 at different times of day. Constructed Langley plots showed roughly linear trends with air mass. The slopes of the best-fit lines were -0.34, -0.32, -0.31 for the blue, green and yellow channels, respectively. The y-intercept provided the expected top-of-the-atmosphere (TOA) signal for each channel.

3. Reference Measurements using Si Detector.

Even though we did not have access to a commercial sun photometer for direct comparison of optical depths, we engineered an alternate solution by using a Si biased detector (Thorlabs, DET36A) integrated with a voltmeter. The voltage signal we measured represents the intensity of the light hitting detector surface. When employed for sun photometry, this scales with direct solar irradiance from the sun. A lens tube was used to narrow the field of view to about 6 degrees. This minimized stray or diffuse light since measurement of direct solar irradiance was desired. The sensor was manually aligned to the sun.

In an effort to test the accuracy of the reference Si sensor we measured the transmittance of 10 neutral density filters (Thorlabs, NEK01) within the yellow, green, and blue pass-bands using the setup. The ND filters had known transmittances from 0.01% to 79.4%. As shown in Fig. S-3, the measured transmittance agreed well with expected values. The standard deviation of the differences (voltmeter trans. – accepted trans.) was 0.028. We

believe this voltmeter should be a reasonably good reference method for optical depth measurements with smartphone.

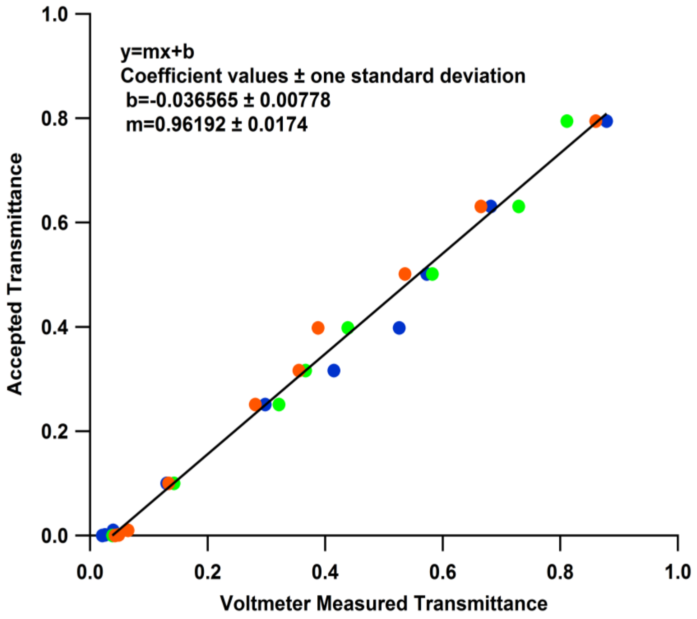

Fig. S-3 Accuracy of reference Si detector / voltmeter for measuring the transmittance of neutral density filters. The different color data points correspond to the yellow, blue, and green sun filters pass-bands.

To determine optical depth with the Si/voltmeter reference device, we also constructed Langley plots (shown in Fig. S-4) to measure the expected top-of-atmosphere signal. Slopes of best fit lines were -0.29, -0.23 and -0.22 for blue, green and yellow channel, respectively.

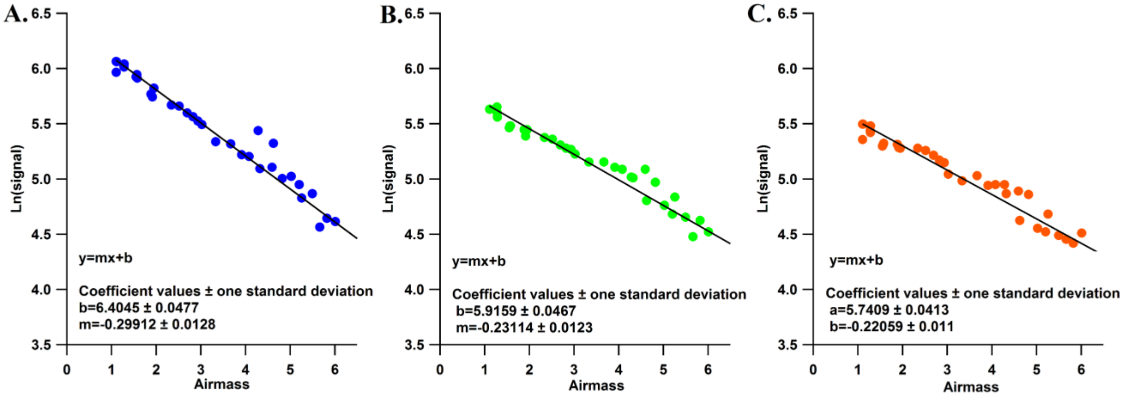

Fig S-4. Langley Plots (ln signal vs. air mass) measured by voltmeter for the blue (A), green (B), and yellow (C) sun filters. The y-intercept describes the signal expected at the top-of-the-atmosphere (TOA) and airmass = 0. Best-fit lines were determined by orthogonal distance regression.
